# Patient HC with developmental amnesia can construct future scenarios

**DOI:** 10.1016/j.neuropsychologia.2011.09.015

**Published:** 2011-11

**Authors:** Niamh C. Hurley, Eleanor A. Maguire, Faraneh Vargha-Khadem

**Affiliations:** a Wellcome Trust Centre for Neuroimaging, Institute of Neurology, University College London, 12 Queen Square, London WC1N 3BG, UK; b Developmental Cognitive Neuroscience Unit, Institute of Child Health, University College London, 30 Guilford Street, London WC1N 1EH, UK

**Keywords:** Scene construction, Episodic memory, Future-thinking, Hippocampus, Autobiographical memory, Semantic memory

## Abstract

Deficits in recalling the past and imagining fictitious and future scenarios have been documented in patients with hippocampal damage and amnesia that was acquired in adulthood. By contrast patients with very early hippocampal damage and developmental amnesia are not impaired relative to control participants when imagining fictitious/future experiences. Recently, however, a patient (HC) with developmental amnesia, resulting from bilateral hippocampal atrophy, was reported to be impaired, thus raising a question about the true nature of event construction in the context of developmental amnesia. Here, we assessed HC on a test of imagination which explored her ability to construct fictitious events or personal plausible future events. Her scenario descriptions were analysed in detail along a range of parameters, using two different scoring methods. HC's performance was comparable to matched control participants on all measures relating to the imagination of fictitious and future scenarios. We then considered why she was reported as impaired in the previous study. We conclude that various features of the previous testing methodology may have contributed to the underestimation of HC's ability in that instance. Patients like HC with developmental amnesia may be successful at future-thinking tasks because their performance is not based on true visualisation or scene construction supported by the hippocampus, but rather on preserved world knowledge and semantic representations.

## Highlights

► Patients with developmental amnesia (DA) have a deficit in recalling the past. ► A number of DA patients have been found to be unimpaired at imagining fictitious/future scenarios. ► Unlike previous DA cases, patient HC was reported to be impaired. ► We tested HC and found her to be comparable to controls at imagining the future. ► Specific features of the previous testing may have underestimated HC's ability.

## 1 Introduction

Patients with bilateral hippocampal damage and amnesia have been found to have deficits not only in recalling the past but also in imagining fictitious and future scenarios (e.g., Andelman, Hoofien, Goldberg, Aizenstein, & Neufeld, 2010; Hassabis, Kumaran, Vann, & Maguire, 2007; Klein, Loftus, & Kihlstrom, 2002; Kurczek et al., 2010; Race, Keane, & Verfaellie, 2011; Rosenbaum, Gilboa, Levine, Winocur, & Moscovitch, 2009; but see Squire et al., 2010, and Maguire & Hassabis, 2011 for a response. Neuroimaging studies have also implicated the hippocampus in imagining fictitious experiences (Hassabis, Kumaran, & Maguire, 2007; Summerfield, Hassabis & Maguire, 2009, Summerfield, Hassabis, & Maguire, 2010) and plausible personal future episodes (e.g., Addis, Wong, & Schacter, 2007; Addis, Pan, Vu, Laiser, & Schacter, 2009; Botzung, Denkova, & Manning, 2008; Szpunar, Watson, & McDermott, 2007).

The study of imagination in amnesic patients, whose damage was acquired in adulthood from a range of aetiologies (including anoxia and limbic encephalitis), has begun to shed some light on the role that the hippocampus might play in supporting autobiographical memory, imagining the future, and also spatial navigation. One study by Hassabis et al. (2007a) found that patients were impaired at imagining fictitious and possible future experiences, with their main problem being an inability to integrate their imagined experiences into a spatially coherent whole, their imagined scenarios being instead spatially fragmented. Based on this it was suggested that perhaps autobiographical memory, imagination and navigation are underpinned by a common core process–the ability to construct coherent scenes (‘scene construction’, Hassabis & Maguire, 2007, 2009). An alternative theory suggests a different account, proposing instead that imagination of the future depends on recombined details of past experiences (‘constructive episodic simulation hypothesis’, Schacter & Addis, 2007).

More recently, the imagination ability of patients with developmental amnesia (DA) has been examined. Such patients sustain selective bilateral hippocampal damage as a result of hypoxic/ischaemic episodes that occur perinatally or early in childhood. They typically have intelligence in the normal range, and relatively preserved semantic memory, but are amnesic for recalling autobiographical events and have impaired spatial navigation (Vargha-Khadem et al., 1997). Maguire, Vargha-Khadem, and Hassabis (2010) found that one such patient, Jon (now an adult), was able to construct fictitious and future experiences when tested with the same scene construction task used by Hassabis et al. (2007a), despite 50% volume loss in each hippocampus (Vargha-Khadem et al., 1997). Similarly, Cooper, Vargha-Khadem, Gadian, and Maguire (2011) used an adaptation of the same test in a group of children (*n* = 21) who had experienced neonatal hypoxia/ischaemia, and consequent bilateral hippocampal damage of varying extent. They too were able to successfully construct fictitious scenarios, despite being impaired at recalling recent autobiographical events. This was the case even for the patients with the most severe memory impairment and the greatest hippocampal volume loss. These results from the developmental patients are in clear contrast to impaired scene construction in patients with adult-acquired hippocampal damage. It has been suggested that a relatively intact semantic memory store and/or some functionality in residual hippocampal tissue may underpin this intact scene construction ability in DA patients (Cooper et al., 2011; Maguire et al., 2010).

Findings to date seem to suggest a difference in the effects of hippocampal pathology on future-thinking depending on whether damage occurs in adulthood or much earlier in life. However, another DA patient, HC (DA6 in Adlam, Vargha-Khadem, Mishkin, & deHaan, 2005; Adlam, Malloy, Mishkin, & Vargha-Khadem, 2009; Case E6 in Vargha-Khadem et al., 2003) was recently reported to be impaired at simulating personal future events (Kwan, Carson, Addis, & Rosenbaum, 2010). The test used differed somewhat from that employed by Cooper et al. (2011), Hassabis et al. (2007a), and Maguire et al. (2010), in that there were fewer trials, a different type of cue was used, there was no spatial coherence measure, and the scoring was focused on internal and external details–a legacy of the test's origins in the autobiographical memory literature (Levine, Svoboda, Hay, Winocur, & Moscovitch, 2002). Thus the imagination findings in HC seem to be at odds with the other DA cases in the literature. The aim of the present study was to investigate this apparent anomaly further.

We did this by testing HC when she visited London. We administered the scene construction test used in our previous studies of adult-acquired hippocampal damage (Hassabis et al., 2007a) and DA (Cooper et al., 2011; Maguire et al., 2010). On the basis of the previous findings we hypothesised that HC would be unimpaired on the task relative to age, sex and IQ matched control participants. If this was the outcome, then it would also be necessary to try and understand why Kwan et al. (2010) came to the opposite conclusion when testing the same patient.

## 2 Methods

### 2.1 Case description

HC is right-handed, female, a native English-speaker, and was 22 years old at the time of testing. She was born prematurely at 32 weeks of gestation, and experienced significant respiratory distress requiring intubation and ventilation as a neonate. After discharge from the hospital, HC achieved her developmental milestones normally. Memory difficulties and motor coordination problems were noted around the age of 4 years, before school entry. Throughout her primary and secondary education, HC attended mainstream schools, successfully graduating from high school; she went on to complete a year in technical college, and is currently working towards a culinary degree.

**Fig. 1:**
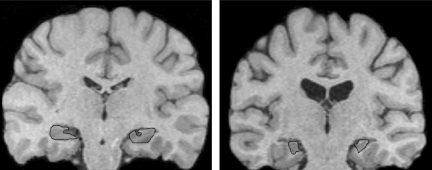
Selected coronal slices of a FLASH MRI scan in an age- and sex-matched healthy control (left), and in HC (right). The hippocampi are outlined in black, and are markedly decreased in volume for HC.

At the age of 14 years 7 months, HC's hippocampal volume reductions in relation to a control group (*n* = 12) of comparable age were 48.1% on the left, and 43.4% on the right (Adlam et al., 2005). Measured again in the summer of 2010 (now aged 22 years), HC's volume reductions relative to the volumes of a large group of healthy controls (*n* = 65) were 45.3% on the left, and 44.8% on the right (see Fig. 1). HC received neuropsychological evaluations in London at the ages of 11, 14, and 22 years. Across the three time periods, including the most recent (see Table 1), various aspects of episodic memory and learning remained severely restricted while standards of intelligence, academic attainments, verbal fluency, working memory, and semantic memory progressed within the normal range, consistent with advances in chronological age (see also Rosenbaum et al., in press; Vargha-Khadem et al., 2003).

### 2.2 Control participants

Fourteen healthy females, who were native English-speakers, and matched to HC on age, IQ (Wechsler Test of Adult reading – WTAR) and handedness also participated in the experiment. The mean age of the control group was 21.5 years (SD 1.56, range 19–24) and mean estimated full-scale IQ was 108.5 (SD 2.14, range 105–112). There was no significant difference between the control participants and HC on either of these background characteristics (age *p* = 0.76; IQ *p* = 0.83). All participants gave informed written consent to participation in the study in accordance with the local research ethics committee.

### 2.3 Task and procedure

A detailed description of the task and the scoring method can be found in Hassabis et al. (2007a) (see also Cooper et al., 2011; Maguire et al., 2010). In brief, each participant was tested individually and the session was recorded digitally for later transcription and scoring of participant responses. The requirements of the task were explained to the participant and examples were given. During this practice session, the experimenter continued until they were satisfied that the participant had fully understood what was required of them. It was also established that the patient could remember the instructions and the cues throughout a construction trial. The scenarios purposely encompassed a variety of different subject matter from the man-made to the natural, and the busy to the isolated to ensure there were no content biases. Each participant completed 10 trials, 7 involving fictitious scenarios (a swimming pool, harbour, library, boardroom, derelict building, circus, and cathedral). In three additional trials we also examined the effect of scenarios that were explicitly self-relevant and potentially plausible in the future (possible event during next weekend, possible Christmas event, possible future meeting with a friend).

For each scenario a short description was read out loud by the interviewer from a prepared script (e.g., “Imagine you are walking along a busy fishing harbour”) and the participant was instructed to vividly imagine the situation suggested by the cue and describe it in as much (multi-modal) detail as possible. Participants were explicitly told not to recount an actual memory or any part of one but rather create something new. A printed text card was placed on the desk in front of the participant containing the cue sentence to act as a reminder if needed. Participants were allowed to continue with their descriptions until they came to a natural end or they felt nothing else could be added. A probing protocol dictated the appropriate use of statements used by the examiner during the session. These mostly took the form of general probes encouraging further description (e.g., “can you see anything else in the scene?”), or asking for further elaboration on a theme introduced by the participant (e.g., “can you describe the fishing boat in more detail?” in response to the participant saying “I can see a small fishing boat gently rocking out in the sea”). It was strictly prohibited for the examiner to introduce any concept, idea, detail or entity that had not already previously been mentioned by the participant. After each scenario, participants were asked to rate their constructions on a number of different parameters (see Section 2.4). At various points during a trial, and prior to the post-scenario ratings, the examiner verified that the participant still recalled the task instructions, the scenario in question, and the scenario she had created.

### 2.4 Scoring

Two scoring methods were employed. The first was that originally developed and employed by Hassabis et al. (2007a), and used in previous studies of patients with early hippocampal damage (Cooper et al., 2011; Maguire et al., 2010). The second was the scoring method used by Kwan et al. (2010) in their previous study of patient HC. By using both methods on the same data we could assess the impact of scoring system on the results.

#### 2.4.1 Hassabis et al. (2007a) scoring

A composite score, the Experiential Index, ranging from 0 to 60, measuring the overall richness of the imagined experience, was calculated from four subcomponents.

*Content*: Each scenario description was segmented into a set of statements. Every statement was then classified as belonging to one of the four main categories: spatial reference, entity presence, sensory description, or thought/emotion/action. Repeated statements, irrelevant details and other tangential information that could not be classified into one of these four categories were excluded. Extensive pilot studies indicated that the production of seven details per category was an optimal reflection of performance while ensuring that those with more circuitous descriptions were not unfairly advantaged. Thus, the score for each details category was capped at a maximum of 7.

*Participant ratings*: Two subjective self-ratings contributed to the Experiential Index, each varying on a scale from 1 to 5: sense of presence (1 – ‘did not feel like I was there at all’; 5 – ‘felt strongly like I was really there’) and perceived salience/vividness (1 – ‘could not really see anything’; 5 – ‘extremely salient’).

*Spatial Coherence Index*: As part of the feedback on each scenario, participants were presented with a set of 12 statements each providing a possible qualitative description of the newly constructed experience. Participants were instructed to indicate the statements they felt accurately described their construction. They were free to identify as many or as few as they thought appropriate. Of the 12 statements, 8 were ‘integrated’ and indicated that aspects of the scene were contiguous (e.g., ‘I could see the whole scene in my mind's eye’) and 4 were ‘fragmented’ and indicated that aspects of the scene were not contiguous (e.g., ‘It was a collection of separate images’). One point was awarded for each integrated statement selected and one point taken away for each fragmented statement. This yielded a score between −4 and +8 that was then normalised around zero to give final Spatial Coherence Index score ranging between −6 (totally fragmented) and +6 (completely integrated). Any construction with a negative Spatial Coherence Index was considered to be incoherent and fragmented.

*Quality judgement*: The final scoring component was the scorer's assessment of the overall quality of the construction. Scorers were instructed to rate how well they felt the description evoked a detailed ‘picture’ of the experience in their own mind's eye. Quality ratings could range from 0 (indicating the construction was completely devoid of details and with no sense of experiencing) to 10 (indicating an extremely rich and highly evocative construction that appeared to emerge from an extremely vivid imagining).

Several other ratings were also taken. After imagining each new experience, participants rated how difficult they found this on a scale from 1 to 5 (1 – very easy … 5 – very difficult). They were also asked to rate on a scale of 1 to 5 its similarity to an actual memory, in whole or in part (1 – nothing at all like any memories … 5 – exactly like a memory).

A series of scenarios (15%) were randomly chosen and scored blindly by a second scorer. To measure inter-rater reliability, the Cronbach's Alpha test of internal consistency was performed on the two raters’ scores, which revealed excellent inter-rater reliability over all sub-components, both with and without the quality judgement included (in both cases *α* = 0.97).

#### 2.4.2 Kwan et al. (2010) scoring

Kwan et al. (2010) used the standard scoring procedure from the autobiographical interview (AI) devised by Levine et al. (2002) when assessing the four personal future events they had HC construct. In order to make a close comparison with Kwan et al. (2010), we took the three of our scenarios that related to personal future events and scored them using the AI method.

Each future scenario was segmented into details, where a detail was defined as a unique occurrence, observation or thought. All details were then categorized as either internal or external. Internal details were those that pertained directly to the central event described by the participant, were specific to time and place, and were considered to reflect episodic experiencing. Internal details were separated into five categories: event, time, place, perceptual and emotion/thought. External details were non-episodic details that were tangential or unrelated to the central event. These included details from other events, semantic facts, repetitions, or metacognitive statements/editorializing. Details for each category were tallied and averaged across the three future scenarios to form internal and external composite scores for each participant.

Each scenario also received qualitative ratings from the scorer for episodic richness (0–6), time (0–3), place (0–3), perception (0–3), and emotion/thoughts (0–3) (see Levine et al., 2002 for descriptions). These scores were tallied and averaged across the three scenarios to determine an overall composite of qualitative ratings (maximum = 18) for each participant. A rating of time integration (0–3) was also assigned to each scene, but was not included in the ratings composite, in line with the Levine et al. (2002) protocol. As with the Hassabis et al. (2007a) scoring method, a series of scenarios (20%) were randomly chosen and scored by a second scorer. To measure inter-rater reliability, the Cronbach's Alpha test of internal consistency was performed on the two raters’ scores, which revealed excellent inter-rater reliability over all components, and for internal, external and ratings separately (in all cases *α* = 0.99).

### 2.5 Data analysis

In order to compare patient HC to the group of 14 controls, we used a modified *t* test (Crawford & Garthwaite, 2002; Crawford & Howell, 1998). This test treats an individual patient as a sample, affording the comparison of the patient and a small control group. All results are two-tailed with a significance threshold of *p* < 0.05.

## 3 Results

### 3.1 Data scored using the Hassabis et al. (2007a) scheme

**Fig. 2:**
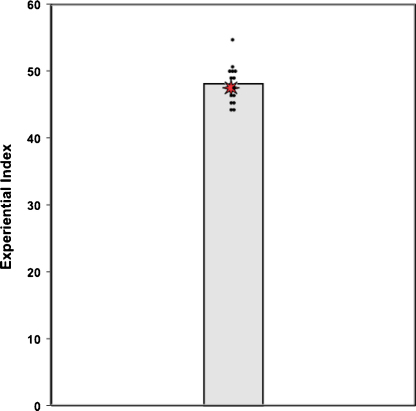
Scores on the Experiential Index (using the Hassabis et al., 2007a scoring method). The data point for each control participant is represented by a black dot. The vertical bar signifies the group mean of the controls. The data point for patient HC is represented by a red star. (For interpretation of the references to colour in this figure legend, the reader is referred to the web version of this article.)

The performance scores for HC and the control group, and statistical comparisons between the two, are summarised in Table 2. There was no significant difference between HC and the controls on the overall Experiential Index. This is illustrated further in Fig. 2, which clearly shows that HC performed within the mid-upper range of the control participants. Similarly, she performed comparably to controls on the sub-component measures of content.

Analysis of uncapped measures of content (i.e., not restricted to seven details per category) also showed that HC's scores were unimpaired in all four categories–spatial references (*t*(13) −0.18, *p* = 0.86), entities present (*t*(13) −1.01, *p* = 0.33), sensory descriptions (*t*(13) −1.15, *p* = 0.27) and thoughts/emotions/actions (*t*(13) −0.63, *p* = 0.54).

HC's overall quality judgement score (given by the scorers) was not significantly different from the controls (see Table 2). When the quality judgement component was excluded from the Experiential Index, the overall score remained comparable to that of the control group (*t*(13) 0.33, *p* = 0.75), showing this subjective scorer rating did not influence the Experiential Index significantly.

**Fig. 3:**
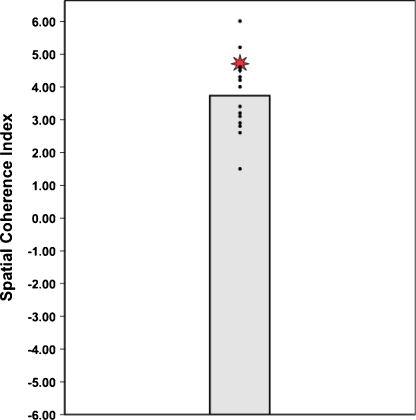
Scores on the Spatial Coherence Index (using the Hassabis et al., 2007a scoring method). The data point for each control participant is represented by a black dot. The vertical bar signifies the group mean of the controls. The data point for patient HC is represented by a red star. (For interpretation of the references to colour in this figure legend, the reader is referred to the web version of this article.)

HC's score on the Spatial Coherence Index was also indistinguishable from controls (see Table 2 and Fig. 3).

**Fig. 4:**
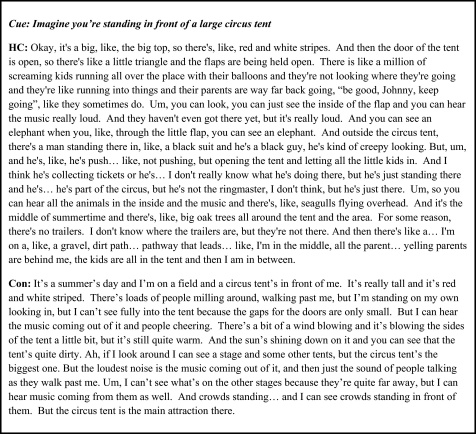
Examples of an imagined fictitious scenario. Representative excerpts with the cue at the top. HC = the patient; con = one of the control participants. Of note, HC has no memory of ever having been to a circus.

There were two types of scenario, fictitious and personal future. Mean Experiential Index scores for the fictitious experiences [HC: 49.69; controls 48.55 (3.66)] were not significantly different (*t*(13) 0.30, *p* = 0.77). This was also the case for the Spatial Coherence Index [HC: 4.57; controls: 3.80 (1.16); *t*(13) 0.64, *p* = 0.53.] Fig. 4 provides excerpts from HC and an example control participant for a fictitious scenario.

**Fig. 5:**
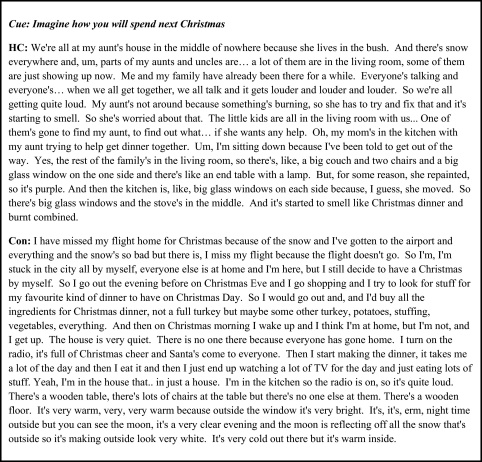
Examples of an imagined personal plausible future scenario. Representative excerpts with the cue at the top. HC = the patient; con = one of the control participants.

Mean Experiential Index scores for the personal future experiences [HC: 42.53; controls 47.07 (3.06)] were also not significantly different (*t*(13) −1.43, *p* = 0.18). This was also the case for the Spatial Coherence Index [HC: 5.00; controls: 3.60 (1.56); *t*(13) 0.87, *p* = 0.40]. Fig. 5 provides excerpts from HC and an example control participant for a personal future scenario.

There was no difference between HC's self-ratings and those of the control participants for perceived salience/vividness, sense of presence, task difficulty and similarity to real memories (see Table 2). The task was rated as easy by HC and controls, and constructed scenarios were also rated by participants, including HC, as being dissimilar to real memories.

### 3.2 Data scored using the Kwan et al. (2010) scheme

**Fig. 6:**
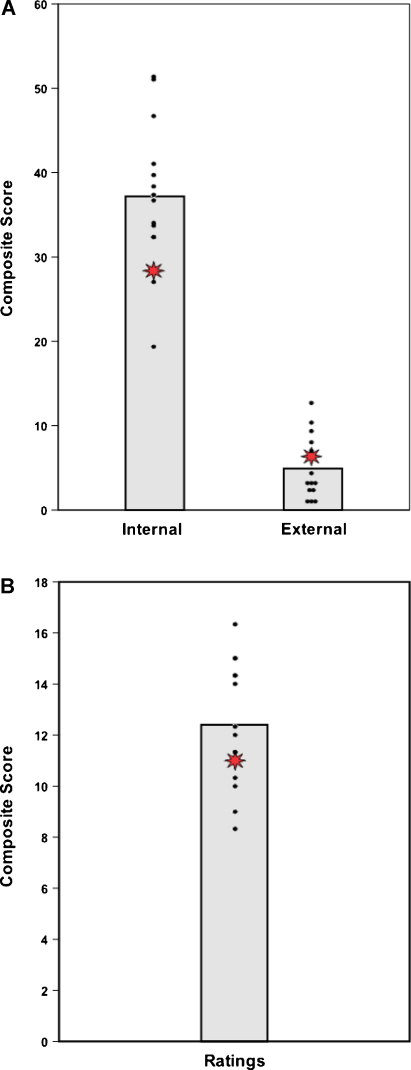
Scores for internal and external details (using the Kwan et al., 2010 scoring method). The three personal future scenarios were also scored using the Kwan et al. method. The data point for each control participant is represented by a black dot. The vertical bar signifies the group mean of the controls. Note that some of the control data points completely overlap. The data point for patient HC is represented by a red star. A. Composite scores for internal and external details. B. Composite scores across qualitative ratings. (For interpretation of the references to colour in this figure legend, the reader is referred to the web version of this article.)

Mean scores and details of the statistical comparisons between HC and the control group on future imagined scenarios using the AI scoring procedure are summarized in Table 3. Analysis revealed that there was no significant difference between HC and the controls on either quantitative (internal and external detail composites, see also Fig. 6A) or qualitative (ratings, see Fig. 6B) scores. HC performed within the lower range of the control participants on internal details, and in the middle range of the controls on external details and on the ratings score.

## 4 Discussion

Previous studies of patients with bilateral hippocampal damage and amnesia have documented impairments in imagining fictitious and future experiences (Andelman et al., 2010; Hassabis et al., 2007a; Klein et al., 2002; Kurczek et al., 2010; Maguire & Hassabis, 2011; Race et al., 2011; Rosenbaum et al., 2009). In contrast to these patients whose hippocampal damage was acquired in adulthood, patients who sustained very early injury to their hippocampi and have developmental amnesia were found to have intact ability to construct scenarios (Cooper et al., 2011; Maguire et al., 2010). However in a recent report on one of the DA patients (HC) in our series (DA6 in Adlam et al., 2005; patient E6 in Vargha-Khadem et al., 2003), Kwan et al. (2010) observed that imagination of future experiences in HC was impaired. Having evaluated HC, we now report that we found her to be unimpaired on measures relating to imagined fictitious events and personal plausible future events. This finding is consistent with previous cases of early hippocampal damage who performed normally, and in contrast to adult-acquired cases of bilateral hippocampal damage who did not. Given this discrepancy in findings when two different research groups evaluated the same patient, the question arises as to the possible reasons for such a difference? Assuming that HC was not having a bad day during testing in Ontario, and a good day during testing in London, then the obvious disparity is in the tests that were employed.

### 4.1 Key features of the two testing protocols

Kwan et al. (2010) used a test with four trials all relating to personal future events, each trial being initiated by a single cue word (e.g., “coffee”). Participants were required to use the cue to help them to generate a scenario. They also had to adhere to another instruction, namely, on some trials they had to create an event that could occur in the next few weeks, and on others, in the next few years. The time limit for each trial was 5 min. The construction of future events was performed as part of a larger task where recall of remote and more recent autobiographical events was tested in a similar manner. The verbal output of the tasks was divided by the scorer into two categories, internal and external details, approximately equivalent to episodic and semantic details. Each event was also given qualitative ratings by the scorer for episodic richness, time, place, perceptual, thought/emotion and time integration. Several ratings were also made by participants concerning vividness, emotionality and personal significance on each trial.

The task we used had ten trials, seven of which involved creation of general fictitious scenarios, and three concerned personal plausible future experiences. For the former, the cues were one sentence long (e.g., “Imagine you are standing in front of a large circus tent”). In the case of the future scenarios, the cues were non-specific (e.g., “Imagine the next time you will meet a friend”/“Imagine something you will be doing this weekend”/“Imagine how you will spend next Christmas”). There was no time limit, although typically no trial lasted beyond 5 min. Scoring was as described in Table 2, where the number of items within four content categories was calculated. There was also a set of participant ratings including vividness (salience), sense of presence, difficulty and similarity to real memories. In addition, participants scored the spatial coherence of their imagined scenario. Finally, the scorer gave an overall assessment (quality judgement) of how well they felt the description evoked a detailed picture of the experience in their own mind's eye.

### 4.2 Possible reasons for the discrepant results

Perhaps the difference in trial number is the reason for the discrepancy between our findings and those of Kwan et al. (2010). It could be argued that with just four trials, they may have lacked sufficient power to sample HC's performance. However, we believe this is an unlikely explanation, because if one considers our three personal plausible future event trials, which are most comparable to the Kwan et al. (2010) task, then we see no impairment in HC compared with controls, thus the same results pertained with ten trials or three trials.

The next obvious difference between the two tasks is how they were scored. Kwan et al.’s (2010) main scoring comprised a categorical distinction between internal and external details, supplemented by a range of scorer judgments. By contrast, we counted up the number of details in four content categories, and had one scorer rating (quality judgement). In our case, the overall results remained the same with or without this subjective scorer rating. Unfortunately Kwan et al. (2010) did not include example transcripts from HC and controls, which would have been helpful in order to examine the data more directly. Nevertheless, when we re-scored our personal future scenario transcripts using the scheme of Kwan et al. (2010) (taken from Levine et al., 2002), while HC was at the lower end of the control range for internal details, there were no statistically significant differences between HC and the control participants for internal details, external details or ratings. Given that the same (unimpaired) result pertained irrespective of the scoring method used suggests this may not be the reason for the disparity between our data and those of Kwan et al. (2010).

Another difference between the two tasks that may be particularly critical is the nature of the cues. Kwan et al. (2010) employed single words as cues, while we used one-sentence cues. Single word cues may force a participant to both construct a scenario and then elaborate upon it. This distinction between event construction and elaboration was initially suggested by Conway, Pleydell-Pearce, and Whitecross (2001); see also Conway, Pleydell-Pearce, Whitecross, and Sharpe (2003), and subsequently adopted by Addis et al. (2007). Construction is described by Kwan et al. (2010) as the creation of a future event, and elaboration as the subsequent imagining of supplementary details. Leading on from this distinction, it could be argued that our one-sentence cues provide the basic scene/scenario and what was then required by HC was the mere elaboration of that scene, thus our cues eschewed the need for actual scene construction. Using this logic it could be concluded that perhaps testing of HC highlights a distinction whereby event construction is impaired in the context of hippocampal amnesia while event elaboration is intact. However, there are a number of problems with this argument.

While the cues for our general fictitious scenarios typically contained a specific spatial context and primary content cues, this was clearly not the case for our future-oriented scenarios. In those trials the cues did not contain any spatial context or any key content cues and were clearly non-specific (e.g., “Imagine something you will be doing this weekend”). Not only does the participant have to generate a specific scenario idea, but she then has to construct it, and subsequently elaborate upon it. Nevertheless HC was still able to perform comparably to the control participants on these non-specific cue trials. Incidentally, if the argument is that provision of specific cues somehow helps to circumvent the construction problem in hippocampal amnesia, then why are patients with bilateral hippocampal damage and amnesia acquired in adulthood so impaired on our task (Hassabis et al., 2007a)? The specific cues for the fictitious scenarios clearly did not help them. Indeed even when they were provided with a specific cue and all of the elements they would need to construct an appropriate scenario, they were unable to do so, their problem instead being an inability to integrate the imagined scenario into a spatially coherent whole (Hassabis et al., 2007a).

Instead, we suggest that the key issue may be with Kwan et al.’s (2010) use of very specific single word cues (e.g., “coffee”). These cues highly constrain the participant, making the task difficult, loading on strategic searching as opposed to event construction (see also Kwan et al., 2010 for more on this). Add to this consideration of two other factors – the additional requirement in the Kwan et al. (2010) study to produce an imagined scenario appropriate to a specific future time period, and the fact that this task was conducted as part of a test of recalling past autobiographical events – and we suggest this may have been too much for amnesic patient HC to keep track of. We believe that Kwan et al.’s (2010) results were possibly influenced by difficulties associated with the cue, and the attendant test context, that any patient with brain injury might find challenging.

### 4.3 Imagination in the context of developmental amnesia

HC was unimpaired at the construction of fictitious and future experiences using our paradigm. This finding is consistent with other reports in the literature of patients with early hippocampal damage (Cooper et al., 2011; Maguire et al., 2010). It is possible that the remnant hippocampal tissue in patients like HC retains some functionality, sufficient to support a form of basic scenario construction, but not enough for the reconstruction of detailed trial unique autobiographical events that are precisely marked in space and time. Future fMRI studies will be needed to examine this issue. Alternatively, patients with developmental amnesia, including HC, who have developed normal intellectual status, and have acquired a wealth of world knowledge, may rely on their semantic memories to construct scenarios. Thus, patients with developmental amnesia may be successful at future-thinking tasks because their performance is not based on true visualisation supported by the hippocampus, but rather on preserved world knowledge and a sense of familiarity with how future events logically unfold. Another DA patient, Jon, described his ability to construct scenarios as something that he had worked on over the years, that is was effortful and did not come automatically or naturally (Maguire et al., 2010). Interestingly, HC was unable to describe exactly how she constructed the scenarios, which was in contrast to her controls who reported two clear strategies, visualising of the initial spatial context and then populating this with relevant detail, or seeing part of the full scene and then mentally scanning around which allowed further aspects of the full scene to come into view. Given that DA patients sustained such early hippocampal damage, well before hippocampal-dependent memory functions had emerged, and have never known what it is like to truly visualise a scene or recreate an event in the mind, then in the absence of any comparator, it is perhaps not surprising that they consider their constructions to be coherent, and rate them as such. They are also very accustomed to relying on their world knowledge and semantic representations day to day, perhaps doing so much more readily than the adult-acquired cases of hippocampal damage, who are acutely aware of the devastating change that has occurred in their ability to imagine scenes pre and post lesion.

To conclude, in this study we attempted to understand why patient HC, with developmental amnesia, was reported to be impaired at imagining the future (Kwan et al., 2010), contrary to other cases of early hippocampal damage reported in the literature (Cooper et al., 2011; Maguire et al., 2010). When we tested HC we found she performed similarly to control participants, a finding that is at variance with the prior report by Kwan et al. (2010) on this patient. We suggest that various features of the Kwan et al. (2010) testing methodology may have contributed to the underestimation of HC's ability in that instance. Future studies will be needed to explore in greater detail the effect on scene construction of different types of cues, test conditions, and strategies in both developmental and adult-acquired amnesia. Overall, our study illustrates that a form of scenario construction may be possible that is not hippocampal-dependent and not based on true visualisation of space and scenes, and this option might become viable/likely when semantic representations of the physical world can be relatively spared as is the case in developmental amnesia. In future studies it will be important to compare different forms of construction, and in particular, to understand any consequences that might arise when the hippocampus is not involved.

## Figures and Tables

**Table 1:** Neuropsychological test scores for patient HC.

Cognitive domains	Measures	HC aged 22 years
*Intelligence*		
Wechsler adult intelligence scale -III (standard scores *X* = 100; SD 15)	Verbal IQ Performance IQ Full scale IQ Verbal comprehension Perceptual organization Working memory Processing speed	105 113 109 103 107 108 125

*Academic attainments*		
Wechsler objective reading dimensions (standard scores *X* = 100; SD 15) Wechsler individual achievement test – II (standard scores *X* = 100; SD 15)	Word reading Word spelling Reading comprehension Mathematical reasoning Numerical operations	103 87 121 102 99

*Semantic knowledge*		
Letter fluency (FAS) (standard scores *X* = 100; SD 15)		115
Category fluency (animal names & boys names) (standard scores *X* = 100; SD 15)		115
British picture vocabulary scale (standard scores *X* = 100; SD 15)	Total score	100
Pyramids & palm trees (raw score/52)	Total score	51

*Attention/executive functions*		
Test of everyday attention (standard scores *X* = 100; SD 15)	Map search (1 min) Map search (2 min) Elevator counting (EC) EC with distraction Visual elevator (accuracy) Visual elevator (time) Elevator counting with reversal Telephone search (time) Telephone search with counting (DT decrement) Lottery	115 130 Normal 115 120 120 115 130 100 75

*Working memory*		
No. of items reproduced	Digit span (forward, backward) Block span (forward, backward)	9,4 6,6

*Long term memory*		
Wechsler memory scale-adults (standard scores *X* = 100; SD 15)	General memory Verbal – immediate recall Verbal – delayed recall Visual – immediate recall Visual – delayed recall Delayed recognition Working memory	49 71 46 71 59 75 105
Rivermead behavioural memory test – extended (raw score/48)	Profile score	10 (impaired)
California auditory verbal learning test (standard scores *X* = 100; SD 15)	Interference Immediate recall Delayed recall Learning trial 1 Learning trial 2 Learning trial 3 Learning trial 4 Learning trial 5	92 40 47 92 92 92 92 77
Design learning test – BMIPB^a^ (raw scores − mean (SD) for age group 16–29 years)	Design learning [total correct lines trials A1–A5, Max 45; *X* = 39.6 (5.2)]	22
	Design learning intrusions [total incorrect lines A1–A5; *X* = 4.1(4.3)]	27
	Design B Interference [total correct lines Max 9; *X* = 6.5 (2.0)]	4
	Immediate recall [total correct lines trial A6, Max 9; *X* = 8.5 (1.2)]	3
	Delayed recall design A (total correct lines trial A7)	0
	Immediate design recall (total correct lines trials A1 + B)	8
Rey-Osterrieth complex figure test (/36; *T*-score: maximum > 80/minimum < 20)	Copy (percentiles) Immediate recall (*T*-score) Delayed recall (*T*-score)	>16 (raw score: 36) <20 (floor level) <20 (floor level)

a Coughlan, Oddy, and Crawford (2007).

**Table 2:** Performance on the imagination task scored using the Hassabis et al. (2007a) method.

	Mean HC	Mean (SD) Controls (*n* = 14)	*t* Value	*p* Value (2-tailed)
Overall richness				
Experiential Index	47.54	48.10 (2.98)	−0.18	0.86
Sub-components				
Content				
Spatial references	4.70	4.81 (1.19)	−0.09	0.93
Entities present	6.90	6.91 (0.11)	−0.09	0.93
Sensory descriptions	6.10	6.87 (0.23)	−3.23	0.007*
Thoughts/emotions/actions	6.40	6.79 (0.33)	−1.14	0.27
Participant ratings				
Perceived salience	4.20	3.76 (0.44)	0.97	0.35
Sense of presence	4.30	3.64 (0.41)	1.56	0.14
Spatial coherence				
Spatial Coherence Index	4.70	3.74 (1.17)	0.79	0.44
Scorer rating				
Quality judgement	6.80	7.51 (0.71)	−0.97	0.35
Other ratings				
Task difficulty	1.50	2.27 (0.43)	−1.73	0.11
Similarity to real memories	1.70	2.04 (0.52)	−0.63	0.54

* Note that the difference between HC and controls on sensory descriptions was driven by one trial. When this trial was removed and the analysis was performed using the other nine trials, there was no significant difference on this (*t*(13) 0.77, *p* = 0.45), or any other measure.

**Table 3:** Performance on the personal future event scenarios scored using the Kwan et al. (2010) method.

	Mean HC	Mean (SD) Controls (*n* = 14)	*t* Value	*p* Value (2-tailed)
*Details*				
Internal	28.33	37.19 (8.75)	−0.98	0.35
External	6.33	4.90 (3.85)	0.36	0.73

*Ratings*	11.00	12.40 (2.47)	−0.55	0.59
